# Supplementary figures and images for: The Drosophila hematopoietic niche assembles through collective cell migration controlled by neighbor tissues and Slit-Robo signaling
Source: bioRxiv. 2024 Oct 15:2024.06.21.600069. Originally published 2024 Jun 25. Preprint. [Version 2] doi: 10.1101/2024.06.21.600069 (PMC11230208; doi:10.1101/2024.06.21.600069)

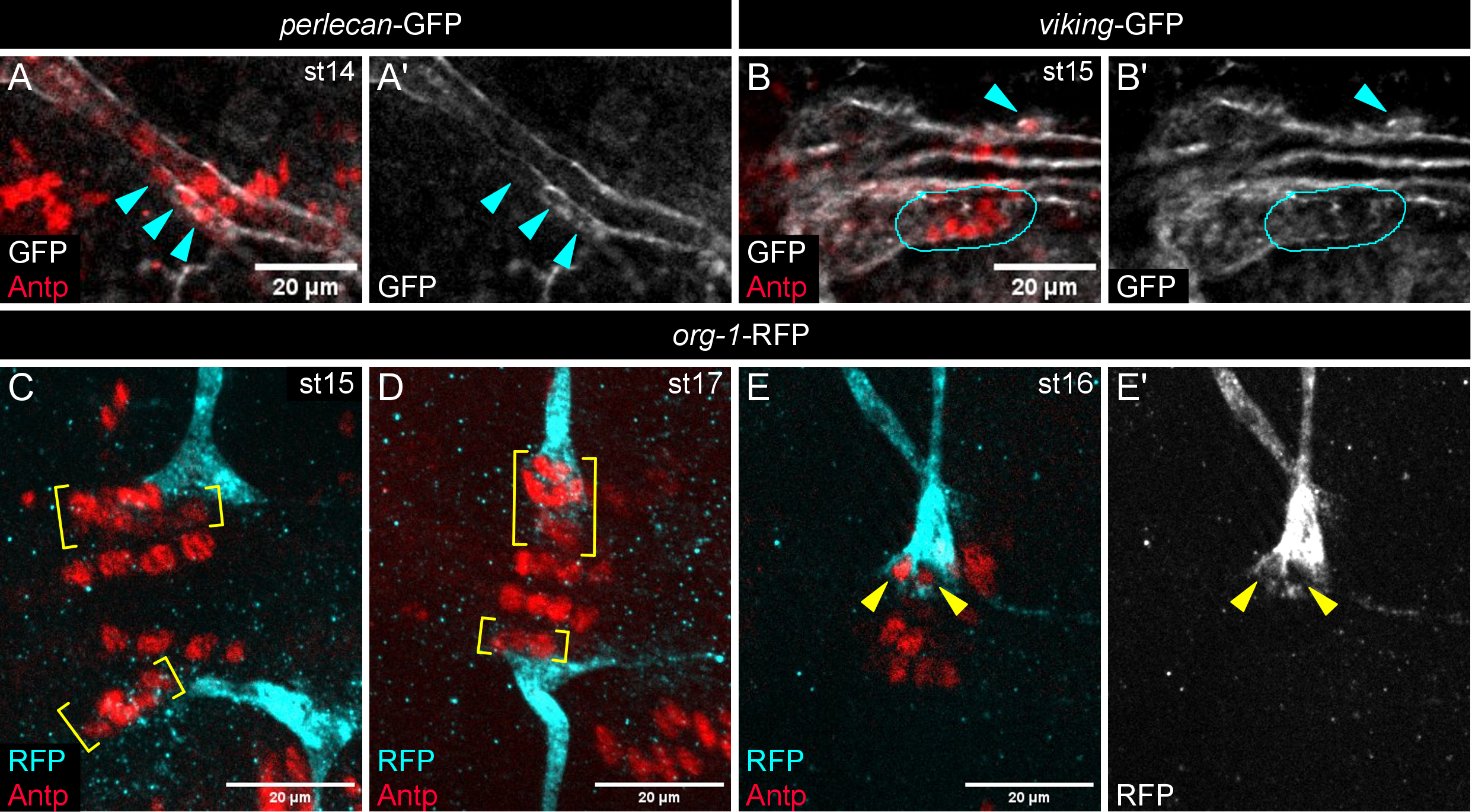

Supplement: Supplement 1 [file media-1.tif]

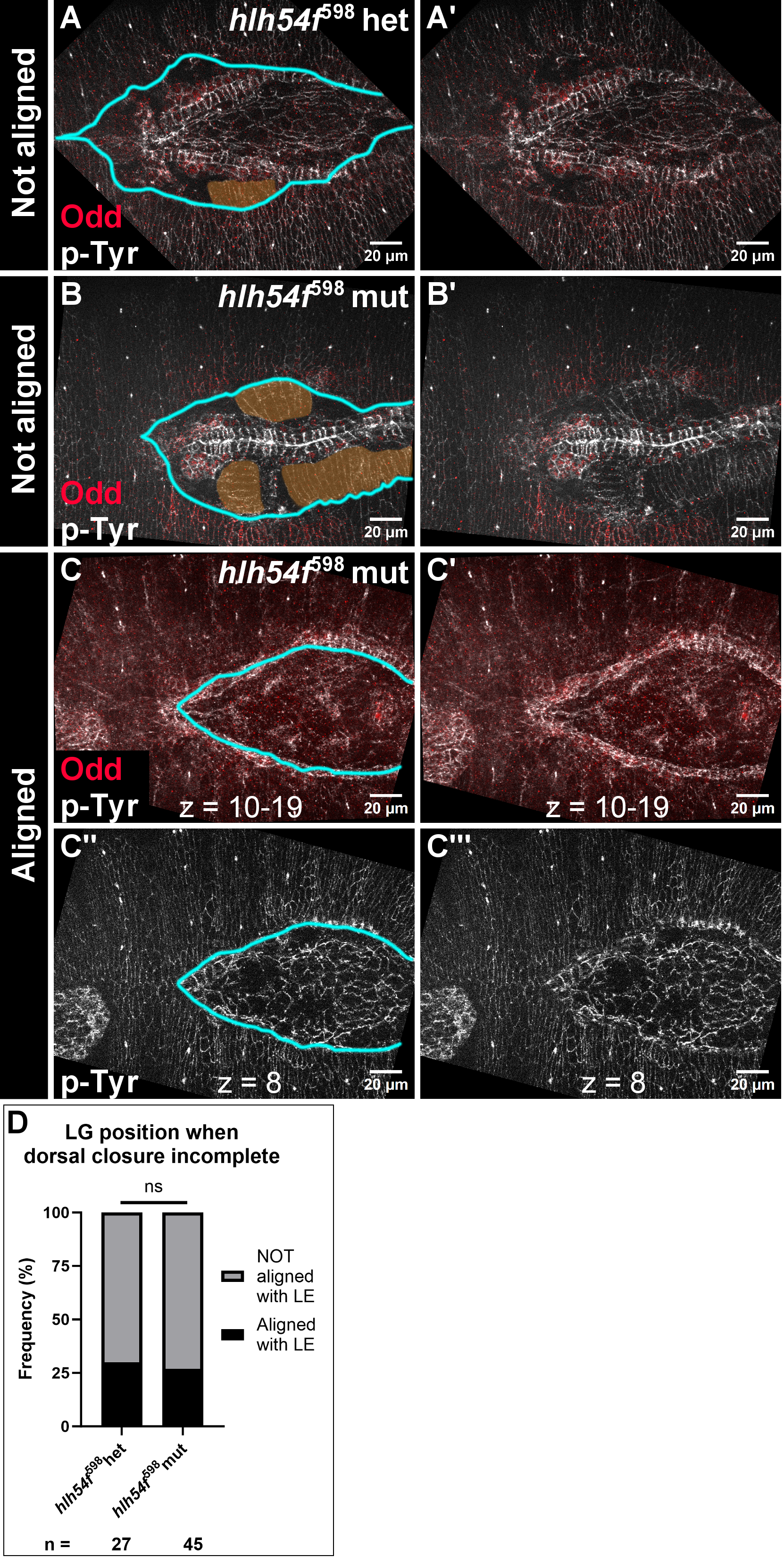

Supplement: Supplement 2 [file media-2.tif]

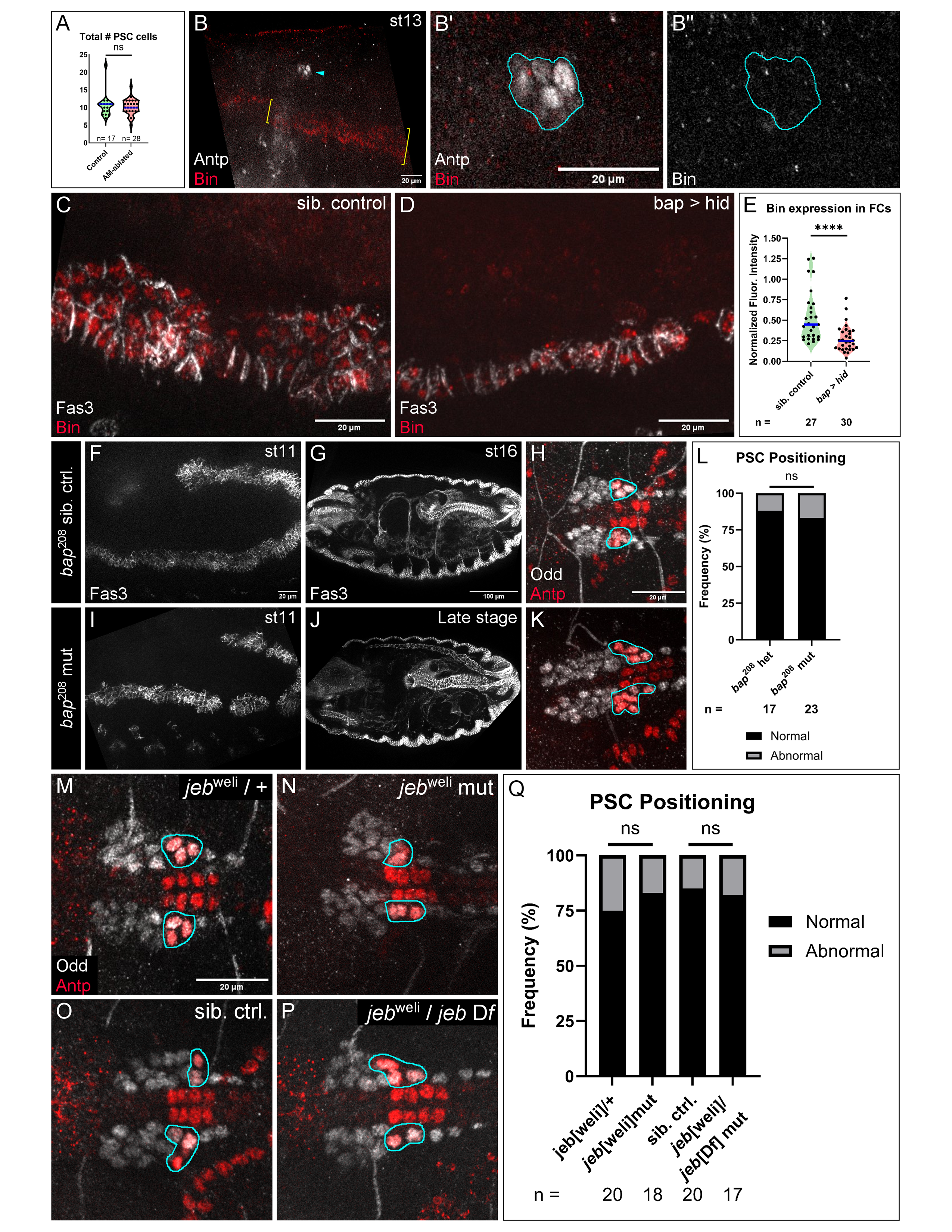

Supplement: Supplement 3 [file media-3.tif]

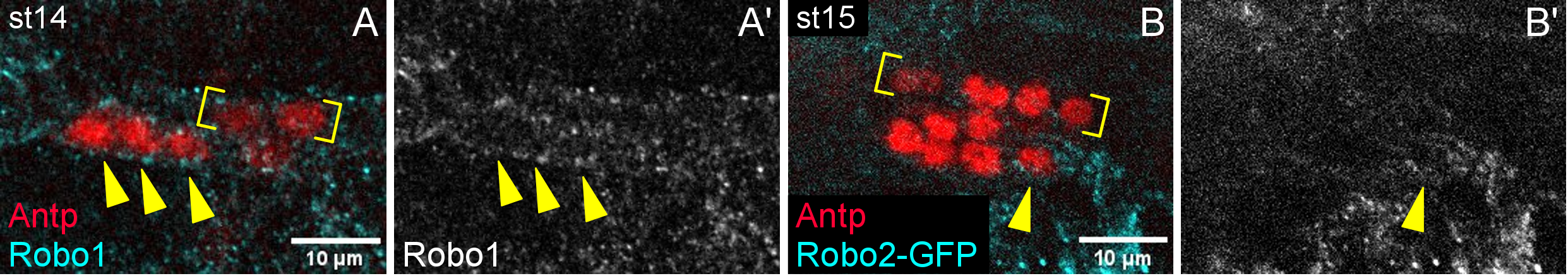

Supplement: Supplement 4 [file media-4.tif]
