# Supplementary material for: The Drosophila hematopoietic niche assembles through collective cell migration controlled by neighbor tissues and Slit-Robo signaling
Source: bioRxiv. 2024 Oct 15:2024.06.21.600069. Originally published 2024 Jun 25. Preprint. [Version 2] doi: 10.1101/2024.06.21.600069 (PMC11230208; doi:10.1101/2024.06.21.600069)
Supplement: Supplement 11 [file media-11.pdf]

Key Resources Table

Reagent type (species) or resource	Designation	Source or reference	Identifiers	Additional information
genetic reagent (*D. melanogaster*)	Antp-GAL4	Emerald and Cohen, 2004	FLYB:FBal0155891	FlyBase symbol: $GAL4^{Antp\text{-}21}$
genetic reagent (*D. melanogaster*)	w^{1118}	Bloomington *Drosophila* Stock Center	BDSC:3605; FLYB:FBal0018186; RRID:BDSC_3605	FlyBase symbol: w^{1118}
genetic reagent (*D. melanogaster*)	Hand-RFP	other		Gift from Georg Vogler
genetic reagent (*D. melanogaster*)	UAS-myr:GFP	Bloomington *Drosophila* Stock Center	BDSC:32200; FLYB:FBti0131976 RRID:BDSC_32200	FlyBase symbol: P{10XUAS-IVS-myr::GFP}su(Hw)attP1
genetic reagent (*D. melanogaster*)	tupAME-GAL4	Bataillé et al., 2020	FLYB: FBtp0142468	FlyBase symbol:P{tup-GAL4.AME-R}<br>Gift from J.L. Frendo
genetic reagent (*D. melanogaster*)	UAS-CD8:GFP	other		Gift from J.L. Frendo
genetic reagent (*D. melanogaster*)	org-1-HN39-RFP	Schaub and Frasch, 2015	FLYB:FBal0276776	FlyBase symbol: $RFP^{org\text{-}1.HN39}$
genetic reagent (*D. melanogaster*)	UAS-grim	Hugo Bellen	FLYB:FBti0154788	Flybase symbol: Dmel\P{UAS-grim.Y}2

genetic reagent (*D. melanogaster*)	*bin*^{R22}	Zaffran and Frasch, 2001	FLYB:FBal0043738	Flybase symbol: Dmel\bin^{R22}
genetic reagent (*D. melanogaster*)	*bin*^{S4}	Zaffran and Frasch, 2001	FLYB:FBal0043739	Flybase symbol: Dmel\bin^{S4}
genetic reagent (*D. melanogaster*)	UAS-hid	Bloomington *Drosophila* Stock Center	BDSC:65403; FLYB:FBti0183136 RRID:BDSC_65403	Flybase symbol: Dmel\P{UAS-hid.Z}2
genetic reagent (*D. melanogaster*)	bap-GAL4	Zaffran and Frasch, 2001	BDSC:91540; FLYB:FBti0214156	Flybase symbol: Dmel\P{bap-GAL4.3}1.1
genetic reagent (*D. melanogaster*)	bap-GAL4	other		gift from Manfred Frasch; Chr: X
genetic reagent (*D. melanogaster*)	tinCΔ4-GAL4	Bloomington *Drosophila* Stock Center	BDSC:92965; FLYB:FBti0216630	Flybase symbol: Dmel\P{tinC-Gal4.Δ4}12a
genetic reagent (*D. melanogaster*)	*slit*^{2}	Bloomington *Drosophila* Stock Center	BDSC:3266 FLYB:FBal0015700	Flybase symbol:Dmel\sli^{2}
genetic reagent (*D. melanogaster*)	*robo1*^{GA285}	other	FLYB:FBal0032588	Gift from Greg Bashaw Flybase symbol:Dmel\robo1^{1}
genetic reagent (*D. melanogaster*)	*robo2*^{1}	Rajagopalan and Dickinson, 2000	FLYB:FBal0121562	Gift from Greg Bashaw Flybase symbol:Dmel\robo2^{1}
genetic reagent (*D. melanogaster*)	*robo2*^{123}	other	FLYB:FBal0123720	Gift from Greg Bashaw Flybase

				symbol:Dmel \robo2^{X123}
genetic reagent (*D. melanogaster*)	UAS-Slit RNAi #1	Vienna *Drosophila* Stock Center	VDRC:v108853 FLYB:FBti0159991	Flybase symbol: Dmel\P{KK100803}VIE-260B
genetic reagent (*D. melanogaster*)	UAS-Slit RNAi #2	Bloomington *Drosophila* Stock Center	BDSC:31468 FLYB:FBal0245521	Flybase symbol:Dmel \sli^{JF01229}
genetic reagent (*D. melanogaster*)	UAS-Robo1 OX	Evans and Bashaw, 2015	BDSC:97240 FLYB:FBal0316479	Flybase symbol:Dmel \robo1 $^{\Delta C.10xUAS.Tag:HA,Tag:SS(wg)}$
genetic reagent (*D. melanogaster*)	UAS-dcr2	Bloomington *Drosophila* Stock Center	BDSC:24650 FLYB:FBti0100275	Flybase symbol:Dmel \P{UAS-Dcr-2.D}2
genetic reagent (*D. melanogaster*)	UAS-dcr2	Bloomington *Drosophila* Stock Center	BDSC:24651 FLYB:FBti0100276	Flybase symbol:Dmel \P{UAS-Dcr-2.D}10
genetic reagent (*D. melanogaster*)	svp-lacZ	Bloomington *Drosophila* Stock Center	BDSC:7314 FLYB:FBti0002862	Flybase symbol: Dmel\P{HZ}svp^{3}
genetic reagent (*D. melanogaster*)	perlecan-GFP	Flytrap; GFP Protein Trap Database	FLYB:FBal0243609	Flybase symbol:Dmel \trol^{ZCL1700}
genetic reagent (*D. melanogaster*)	viking-GFP	Buszczak and Spradling, 2007	FLYB:FBal0211825	Flybase symbol:Dmel \vkg^{CC00791}
genetic reagent (*D. melanogaster*)	*bap*^{208}	Bloomington *Drosophila* Stock Center	BDSC:91539 FLYB:FBal0034201	Flybase symbol:Dmel \bap^{208}

genetic reagent (*D. melanogaster*)	*jeb*^{weli}	Stute and Holz, 2004	FLYB:FBal0159133	Flybase symbol:Dmel\jeb^{weli}
genetic reagent (*D. melanogaster*)	*jeb* Df	Bloomington *Drosophila* Stock Center	BDSC:26551 FLYB:FBab0045764	Flybase symbol:Df(2R)BSC699
genetic reagent (*D. melanogaster*)	robo2-GFP	Bloomington *Drosophila* Stock Center	BDSC:61774 FLYB:FBal0265307	Flybase symbol:Dmel\robo2^{MI04295}
antibody	anti-Antp (Mouse monoclonal)	Developmental Studies Hybridoma Bank	Cat#:8C11, RRID:AB_528083	IF(1:50)
antibody	anti-Odd skipped (Rabbit polyclonal)	Ward and Skeath, 2000		IF(1:400); gift from James Skeath
antibody	anti-GFP (Chick polyclonal)	Aves labs	Cat#:GFP-1020 RRID:AB_2307313	IF(1:1500)
antibody	anti-Fas3 (Mouse monoclonal)	Developmental Studies Hybridoma Bank	Cat#:7G10 RRID:AB_528238	IF(1:50)
antibody	anti-Mef2 (Rabbit polyclonal)	Developmental Studies Hybridoma Bank	Cat#:Mef2 RRID:AB_2892602	IF(1:1000)
antibody	anti-Slit (Mouse monoclonal)	Developmental Studies Hybridoma Bank	Cat#:C555.6D RRID:AB_528470	IF(1:200); gift from Greg Bashaw

antibody	anti-LacZ (Chick polyclonal)	Abcam	Cat#:ab9361 RRID:AB_307210	IF(1:1000)
antibody	anti-RFP (Rabbit polyclonal)	Abcam	Cat#:ab62341 RRID:AB_945213	IF(1:1000)
antibody	anti-Bin (Rabbit polyclonal)	other		IF(1:100); gift from Eileen Furlong
antibody	anti-Robo1 (Mouse monoclonal)	other		IF(1:200); gift from Greg Bashaw
antibody	anti-Odd skipped (Guinea pig polyclonal)	other		IF(1:1200); gift from John Reinitz
chemical compound, drug	Paraformaldehyde	Electron Microscopy Sciences	Cat#:15710	
chemical compound, drug	Propyl-gallate	Sigma Aldrich	PubChem Substance ID:24898394; SKU:P3130; CAS Number:121-79-9	
chemical compound, drug	Normal Donkey Serum	Jackson ImmunoResearch Labs Inc	Cat#:017-000-121 RRID:AB_2337258	
chemical compound, drug	Ringer’s solution	other		Recipe from https://doi.org/10.1242/dev.125.15.2781

chemical compound, drug	Triton X-100	MilliporeSigma	CAS Number: 9036-19-5	
software, algorithm	FIJI	ImajeJ	RRID:SCR_002285	http://fiji.sc
software, algorithm	Photoshop	Adobe	RRID:SCR_014199	https://www.adobe.com/products/photoshop.html
software, algorithm	Prism	Graphpad	RRID:SCR_002798	v9.0.0-v10.0.0
software, algorithm	Axio-Vision Imaging Software	Zeiss		v4.8.1
software, algorithm	VisiView	Visitron		
software, algorithm	Metamorph Microscopy Automation and Image Analysis Software	Leica		v7.8.40
other	63x / 1.2 NA water immersion objective	Leica		
other	60x / 1.3 NA silicone immersion objective	Olympus		
other	AxioCam HRm	Zeiss		
other	40x / 1.2 NA water immersion objective	Zeiss		
other	20x / 0.8 NA objective	Zeiss		

other	M165FC	Leica		
other	Achromat 1.6x objective	Leica		
other	GFP Filter set ET470/40x; ET525/50m	Leica		
other	mCherry Filter set ET560/40x; ET630/75m	Leica		
other	pco.edge 4.2 bi sCMOS	PCO		
other	Cell Center Stockroom (Penn)	other	RRID:SCR_022399	
other	CDB Microscopy Core (Penn)	other	RRID:SCR_022373	
